# The seasons within: a theoretical perspective on photoperiodic entrainment and encoding

**DOI:** 10.1007/s00359-023-01669-z

**Published:** 2023-09-02

**Authors:** Christoph Schmal

**Affiliations:** Institute for Theoretical Biology, Humboldt-Universität zu Berlin, Philippstr. 13, 10115 Berlin, Germany

**Keywords:** Circadian clock, Seasonality, Photoperiodic encoding, Synchronization, Coupling, Mathematical modeling

## Abstract

Circadian clocks are internal timing devices that have evolved as an adaption to the omnipresent natural 24 h rhythmicity of daylight intensity. Properties of the circadian system are photoperiod dependent. The phase of entrainment varies systematically with season. Plastic photoperiod-dependent re-arrangements in the mammalian circadian core pacemaker yield an internal representation of season. Output pathways of the circadian clock regulate photoperiodic responses such as flowering time in plants or hibernation in mammals. Here, we review the concepts of seasonal entrainment and photoperiodic encoding. We introduce conceptual phase oscillator models as their high level of abstraction, but, yet, intuitive interpretation of underlying parameters allows for a straightforward analysis of principles that determine entrainment characteristics. Results from this class of models are related and discussed in the context of more complex conceptual amplitude–phase oscillators as well as contextual molecular models that take into account organism, tissue, and cell-type-specific details.

## Introduction

Circadian clocks are complex systems that integrate different scales of spatio-temporal organization to plastically cope with varying environmental demands in a daily and seasonally changing world. Interlocked transcriptional-translational negative feedback loops are a common design principle underlying single cellular rhythm generation across different species, such as *Neurospora crassa*, plants, insects, and mammals. Such single cellular oscillators coordinate at the tissue and organ level to ensure a proper system level functioning of circadian physiology (Micklem and Locke 2021). A functioning circadian clockwork has been shown to provide an adaptive advantage across different kingdoms of life, ranging from unicellular cyanobacteria to multicellular plants and mammals (Ouyang et al. 1998; Dodd et al. 2005; Spoelstra et al. 2016). In turn, circadian disruption has been linked to adverse health effects, such as an increased risk for cancer, cardiovascular diseases, or mood disorders (Savvidis and Koutsilieris 2012; Crnko et al. 2019).

Stable entrainment of the circadian system to the 24 h rhythms of environmental zeitgeber signals, such as light–dark or temperature cycles, is essential for the proper alignment of physiological processes around the solar day and is thus under evolutionary selection. Intrinsic clock properties and external environmental factors that vary with season and latitude determine at which time of day physiological processes are executed (Hut et al. 2013; Bordyugov et al. 2015). In addition to such zeitgeber- and oscillator-dependent tuning of the phase of entrainment, it has been shown that the circadian system plastically changes in response to previously applied entraining cues such as changing periods or photoperiods (Pittendrigh and Daan 1976a).

In the following sections, we describe general principles of circadian entrainment as well as synchronization or self-entrainment in ensembles of coupled clocks. We extend these concepts toward the entrainment under seasonal conditions and corresponding network re-organizations that have been proposed to underlie photoperiodic encoding within the mammalian core pacemaker. In each section, exemplary intuitive conceptual models are carefully introduced in detail before reviewing more complex approaches as well as detailed contextual molecular models.

## Materials and methods

### Kuramoto model with a bimodal frequency distribution

A system of *N* coupled phase oscillators whose dynamical evolution is given by

1 $$\begin{aligned} {\dot{\theta }}_i(t) = \omega _i + \sum _{i=1}^N K_{ij}(\theta _j-\theta _i) \end{aligned}$$

is known as a Kuramoto model; see also Eq. (14) of the *Main text*. In the section Photoperiodic encoding through network re-organizations, we assume a functional separation of the *N* oscillators into two groups or communities representing, e.g., the core and shell part of the mammalian core pacemaker, the suprachiasmatic nucleus (SCN). In general, the communities can be of different size as described by the fractions $p \in [0,1]$ and $(1-p)$, respectively, with different mean frequencies $\omega _1$ and $\omega _2$ as well as different frequency spreads (or scale factors) $\gamma _1$ and $\gamma _2$ as given by a bimodal Cauchy–Lorentz distribution

2 $$\begin{aligned} g(\omega ) = \frac{p \, \gamma _1}{\pi \left[ (\omega - \omega _1)^2 + \gamma _1^2 \right] } + \frac{(1-p) \, \gamma _2}{\pi \left[ (\omega - \omega _2)^2 + \gamma _2^2 \right] } \quad . \end{aligned}$$

Using the Ott–Antonsen approach (Ott and Antonsen 2008, 2009), the temporal evolution of the communities order parameters $R_1(t)$ and $R_2(t)$ as well as the phase difference between the clusters $\Delta \varphi (t)=\varphi _2(t)-\varphi _1(t)$ under the assumption of identical intra- and inter-community coupling strength $K_{ij}=K$ reads as

3 $$\begin{aligned} {\dot{R}}_1&= - \gamma _1 R_1 + \frac{K}{2} (1-R_1^2)(pR_1+qR_2\cos (\Delta \varphi )) \end{aligned}$$

4 $$\begin{aligned} {\dot{R}}_2&= - \gamma _2 R_2 + \frac{K}{2} (1-R_2^2)(qR_2+pR_1\cos (\Delta \varphi )) \end{aligned}$$

5 $$\begin{aligned} \Delta {\dot{\varphi }}&= \omega _2 - \omega _1 - \frac{K}{2} \sin (\Delta \varphi ) \frac{pR_1^2 + qR_2^2+R_1^2 R_2^2 }{R_1R_2}.\end{aligned}$$

For the sake of simplicity, we assume in Fig. 5 that both communities are of equal size $p=1-p=0.5$ and have an identical frequency spread $\gamma _1=\gamma _2=\gamma$. Under such conditions, one can focus to solutions satisfying the symmetry condition $R(t):=R_1(t)=R_2(t)$, such that above equations facilitate to

6 $$\begin{aligned} \dot{R}&= \frac{K}{4} R \left( 1 - \frac{4 \gamma }{K} - R^2 + (1-R^2) \cos (\Delta \psi )\right) \end{aligned}$$

7 $$\begin{aligned} {\dot{\psi }}&= \Delta \omega - \frac{K}{2} (1+R^2) \sin (\Delta \psi ) \end{aligned}$$

with $\Delta \omega = \omega _2 - \omega _1$, being in line with results of Martens et al. (2009).

### Numerical solutions

Numerical simulations underlying Figs. 4b and 5f have been obtained by integrating Eq. (14) via the odeint function of the SciPy package. Bifurcation diagrams based on Eqs. (6, 7) as depicted in Fig. 5e are obtained via the XPP-AUTO package as previously described (Schmal et al. 2014), using the parameters $Ntst = 150$, $N\max = 20,000$, $Dsmin = 0.0001$, and $Dsmax = 0.0005$.

### Seasonal entrainment

#### Conceptual models explain complex data

*Konopka and Benzer* were the first who discovered a single-gene mutation that affects circadian free-running rhythms in *Drosophila melanogaster* (Konopka and Benzer 1971), leading to a new era of molecular genetics in chronobiology that eventually revealed the molecular constituents of circadian clocks across various organisms, such as cynobacteria, *Neurpospora crassa*, *Arabidopsis thaliana*, *Drosophila melanogaster*, as well as mammals (Bell-Pedersen et al. 2005). Even long before the molecular cogs and levers of the regulatory feedback loops underlying circadian rhythm generation have been found, conceptual oscillator models have been developed and used to understand circadian behavior and photoperiodic responses (Wever 1964; Pavlidis 1967; Winfree 1967). Such conceptual or generic oscillator models do not consider molecular details specific to certain organisms, tissues, or cell types but rather focus on general oscillator properties and their potential to explain observed experimental data (Roenneberg et al. 2008).

##### Phase oscillator models

One of the most abstract and simple conceptual models are phase oscillators. The only variable used to describe the circadian clock dynamics in this class of models is the phase of its oscillation $\theta (t)$ essentially evolving between 0 and $2\pi$. By this, we tacitly assume that the clock self-sustains its oscillation with a robust period $\tau$ or angular velocity $\omega =\frac{2\pi }{\tau }$. *Yoshiki Kuramoto* introduced an intuitive way to describe the interaction between a given oscillator $\theta (t)$ and a second oscillator $\varphi (t)$ by means of a sinusoidal coupling term

8 $$\begin{aligned} \frac{d\theta (t)}{dt} = \omega + z \sin (\varphi (t) - \theta (t)), \end{aligned}$$

such that oscillator $\theta (t)$ slows down in case its phase advances the second oscillator $\varphi (t)$ and speeds up in case it is delayed compared to $\varphi (t)$ as the term $\sin (\varphi (t) - \theta (t))$ in (8) becomes negative or positive, respectively (Kuramoto 1975, 2003).

**Fig. 1:**
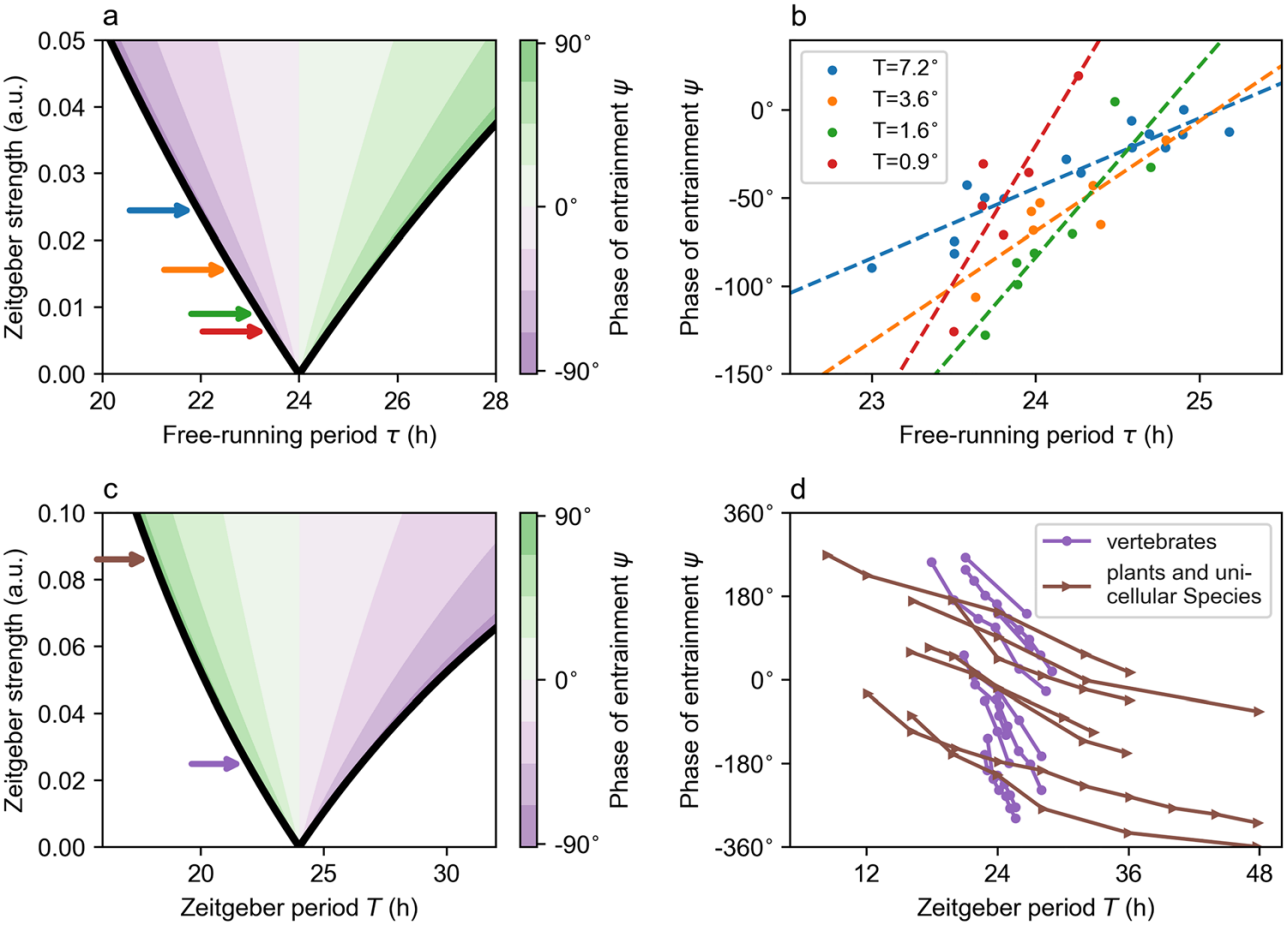
Weak zeitgebers or strong clocks lead to a large phase variability.** a** Arnold tongue based on phase oscillator model (9) in the parameter plane spanned by the internal free-running period $\tau$ and the amplitude or strength *z* of the external zeitgeber signal. Color-coded values depict the phase of entrainment $\psi$ as given by Eq. (11).** b** Experimentally obtained entrainment phases in dependence of the intrinsic free-running period $\tau$ for ruin lizards subject to temperature cycles of different amplitude, i.e., zeitgeber strength. Data have been extracted from Fig. 5 of (Hoffmann 1969) via the WebPlotDigitizer software (Rohatgi 2022).** c** Arnold tongue in the parameter plane spanned by the period *T* and amplitude or strength *z* of the external zeitgeber signal. Color-coded values depict the phase of entrainment $\psi$ as given by Eq. (11).** d** Experimentally obtained entrainment phases $\psi$ for different species subject to entrainment cycles of different external zeitgeber period *T*. Species have been categorized into vertebrates (purple lines) as well as plants and unicellular species (brown lines). Please refer to the original publication (Aschoff and Pohl 1978) for the detailed description of the investigated animals and entrainment properties. Data have been extracted from Fig. 2 of (Aschoff and Pohl 1978) via the WebPlotDigitizer software (Rohatgi 2022)

Even such a simple model allows to explain a variety of experimental results and can help to better understand properties of the circadian clock. Assuming that a circadian clock with period $\tau$ and described by phase variable $\theta (t)$ is driven by an external zeitgeber $\varphi (t)$ of period *T*, the dynamical evolution of the phase difference $\psi (t)=\varphi (t)-\theta (t)$ between the zeitgeber and clock phase is governed by the well-known *Adler* equation

9 $$\begin{aligned} \frac{d\psi (t)}{dt}=\Delta \omega - z \sin (\psi (t)), \end{aligned}$$

where $\Delta \omega = \frac{2 \pi }{T} - \frac{2 \pi }{\tau }$ is the difference of the angular velocity of the zeitgeber and internal clock and *z* is the effective zeitgeber strength. Here, we tacitly assumed that there is no feedback from the clock to the zeitgeber, such that the entrainment cue can be described via $\frac{d\varphi (t)}{dt}=\frac{2 \pi }{T}$, with *T* being the zeitgeber period. From (9), it follows that the circadian clock is only able to entrain to the zeitgeber signal for small enough frequency detunings ($\Delta \omega$) or high enough zeitgeber strengths (*z*) given by the condition

10 $$\begin{aligned} \left\| \frac{\Delta \omega }{z} \right\| < 1. \end{aligned}$$

The range of periods for which the internal clock entrains to the external zeitgeber is termed entrainment range. For a given zeitgeber period, e.g., $T=24$h, the entrainment range generally increases for increasing zeitgeber strength *z*, leading to a wedge shaped entrainment region in the $\tau$-*z* parameter plane, known as the *Arnold* tongue (Fig. 1a). For combinations of free-running periods $\tau$ and zeitgeber strength *z* that lie within the *Arnold* tongue, the internal clock and external zeitgeber signal oscillate with a common period (frequency-locking) and adopt a stable phase relationship $\psi$ (phase-locking). This phase of entrainment $\psi$ is of fundamental importance for the proper alignment of physiological processes around the solar day and thus under evolutionary selection.

Our conceptual phase oscillator model (9) predicts that the dependency of the phase of entrainment $\psi$ on zeitgeber strength *z* and the frequency detuning $\Delta \omega$ is given by

11 $$\begin{aligned} \psi ^{\star }=\arcsin \left( \frac{\Delta \omega }{z}\right) . \end{aligned}$$

Thus, for any given zeitgeber intensity *z*, the phase of entrainment can vary only by 180° with respect to changes of $\tau$ or *T*. *Rütger Wever*, a pioneer in mathematical modeling of the circadian system, described such 180° rule already in 1964 for a conceptual oscillator model adopted from electrical engineering, the Van der Pol oscillator, that has been originally developed to describe oscillations in electrical circuits employing vacuum tubes (Wever 1964). The 180-degree-rule predicts a smaller phase variability with respect to variations of the intrinsic free-running period $\tau$ for increasing zeitgeber strength due to larger entrainment ranges, i.e., phases are less compressed (see color-coded area in Fig. 1a. This is in line with results from early entrainment experiments by *Klaus Hoffmann*, showing that entrainment ranges increase, while the phase variability decreases with an increasing zeitgeber amplitude (strength) for the ruin lizard *Lacerta sicula* subject to temperature cycles of $T=24$h period (Hoffmann 1969), compare Fig. 1b and corresponding arrows in Fig. 1a. Positions of arrows in Fig. 1a, i.e., the zeitgeber strength *z* estimated from the experimental data, have been determined by linear regressions (*dashed lines*) in Fig. 1b.

The resistance of a self-sustained oscillator to entrain to a certain zeitgeber signal can be used to define strong and weak clocks. While strong clocks are characterized by relatively small entrainment ranges and, thus, large phase variabilities with respect to changes in *T*, weak clocks exhibit broad entrainment ranges and small phase variabilities, corresponding to small and large zeitgeber strengths *z* in Fig. 1c. Along these lines, entrainment experiments can be used to categorize circadian pacemakers into weak and strong clocks and to infer internal oscillator properties of a given organism, tissue, or cell type of interest. *Aschoff* and *Pohl* summarized the entrainment behavior of 19 different species subject to entrainment cues of different zeitgeber period *T* (Aschoff and Pohl 1978). By comparing the dependency of the entrainment phase $\psi$ to changes in *T* (i.e., the slope of curves in Fig. 1d) with results from our conceptual model (Fig. 1c), it turns out that vertebrate clocks rather behave like relatively strong clocks (Fig. 1c, purple arrow), while clocks of plants and unicellular species behave more like weak oscillators (Fig. 1c, brown arrow). Along these lines, above-described $180^\circ$ rule has been used to show that strong oscillators like the vertebrate clock with a high phase variability are able to translate a narrow distribution of internal free-running periods $\tau$ in a population with standard deviations of as little as $\sigma =0.2\hbox {h}$ for humans (Duffy et al. 2011) into the experimentally found large spread of human chronotypes which can be related to a large spread in the distribution of entrainment phases $\psi$ (Roenneberg et al. 2004; Granada et al. 2013; Schmal et al. 2020). A similar reasoning has been used to argue that the weak circadian clocks as observed for organisms living at high latitudes such as certain *Drosophila* strains (Beauchamp et al. 2018) or rain deer (van Oort et al. 2005) could be an adaptive advantage as weak oscillators are able to entrain better under extreme photoperiodic conditions such as long summer days or long winter nights in comparison to strong clocks (Vaze and Helfrich-Förster 2016). Analogously to a weaker circadian clock, entrainment can be also facilitated by increasing an organisms light sensitivity as suggested in a comparative study of a northern and southern line of the parasitoid wasp *Nasonia vitripennis* (Floessner et al. 2023).

##### Broad applicability of phase oscillator models

The conceptual phase oscillator approach described in the previous section solely relies on the assumption that oscillators exhibit self-sustained oscillations and that interactions between clocks are weak in a way that amplitude effects can be neglected and the overall system dynamics can be adequately described by its phase of oscillation. Due to the general validity of these assumptions among many systems, the phase oscillator approach has been applied to a plethora of physical, chemical, and biological systems, such as synchronizing fireflies, frog choruses, or the crowd synchronization of pedestrians on London’s Millennium bridge (Ermentrout and Rinzel 1984; Strogatz et al. 2005; Ota et al. 2020), to name a few.

#### Entrainment under varying photoperiods

**Fig. 2:**
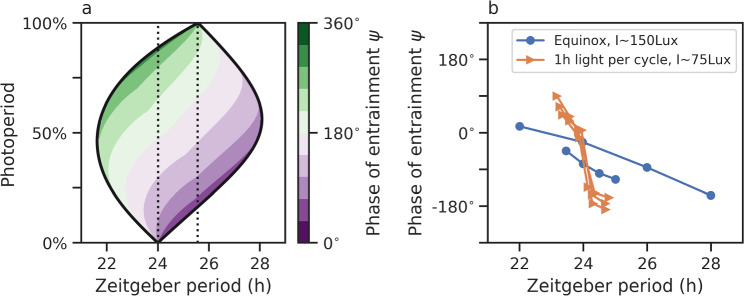
Arnold onions capture essential features of seasonal entrainment.** a** Entrainment regions adopt an onion-shaped geometry in the photoperiod-zeitgeber period parameter plane. The tilt of the Arnold onion can be explained by Aschoff’s rule, i.e., the difference between the internal free-running period under constant darkness (photoperiod of $0\%$) and constant light (photoperiod of $100\%$), depicted by vertical dashed lines. Phases of entrainment $\psi$ are color-coded within the region of entrainment.** b** Experimentally obtained entrainment phase $\psi$ in dependence of the zeitgeber period *T* for the golden hamster *Mesocricetus auratus* subject to light–dark cycles with equinoctial (blue) and extremely short (orange) photoperiods. Data have been extracted from Fig. 3 of (Aschoff and Pohl 1978) via the WebPlotDigitizer software (Rohatgi 2022)

So far, we discussed general principles of entrainment under the assumption of symmetric zeitgeber cues with equal durations of day and night. Due to the tilt of the Earth’s rotation axis with respect to its orbit around the Sun, properties of zeitgeber signals such as the photoperiod of light–dark cycles depend on latitude and season. In Schmal et al. (2015), the concept of Arnold tongues (Fig. 1a, c was extended to account for photoperiodic entrainment, i.e., to zeitgeber cycles of varying daylengths. Since pure phase descriptions as given by Eqs. (8, 9, 10, 11) are unable to directly account for amplitude-dependent effects on entrainment and phase resetting (Lakin-Thomas et al. 1991; Ananthasubramaniam et al. 2020), we use a conceptual amplitude-phase oscillator model

12 $$\begin{aligned} \frac{dr}{dt}&= \lambda r (A-r) \end{aligned}$$

13 $$\begin{aligned} \frac{d \phi }{dt}&= \frac{2\pi }{\tau } \end{aligned}$$

also known as Poincaré oscillator (Glass and Mackey 1988), instead. In radial coordinates as given by Eqs. (12 and 13), variables *r* and $\phi$ denote the time-dependent (instantaneous) amplitude and phase of the internal clock, respectively, while parameters *A*, $\tau$ and $\lambda$ conveniently describe properties of the internal clock such as the steady-state amplitude, period, and radial relaxation rate which can differ and be related to specific organisms, tissues, or cell types. The resulting entrainment regions in the photoperiod and zeitgeber period parameter plane have their largest entrainment range at the equinox and taper toward the internal clocks free-running period $\tau$ under constant darkness and constant light (Fig. 2a). The tilt of this *Arnold Onion* is given by Aschoff’s rule, i.e., the difference between $\tau$ under constant darkness and constant light, with the internal period under constant light being typically shorter or longer compared to the period under constant darkness in day-active animals and plants or night-active animals, respectively (Aschoff 1960; Pittendrigh 1960). A complementary theoretical treatise to explain the emergence and properties of Arnold onions using a pure phase oscillator description as given by Hoveijn (2016) connects these results with the mathematical approach of the previous section.

Again, such a straightforward conceptual model is able to explain a variety of experimental results on photoperiodic entrainment. For the model assumptions underlying Fig. 2a, the 180° rule holds true within the entrainment range at a given fixed photoperiod, analogous to the observation for pure oscillators in Fig. 1. From this, it follows that the phase variability with respect to changes in zeitgeber period *T* is lowest under equinoctial conditions and increases with increasing or decreasing photoperiods as experimentally observed for golden hamster entrained to light–dark cycles of different photoperiods; compare Fig. 2b. Another prediction from Fig. 2a is that a large tilt of the Arnold onion as given by Aschoff’s rule can lead to a situation where the internal clock might be able to entrain to zeitgeber signals of short but not of long photoperiods or vice versa. This phenomenon can explain why the drinking behavior of squirrel monkeys (*Saimiri sciureus*) synchronizes to 24 h light–dark schedules of extremely short photoperiods but not to those longer than 21 h (Schmal et al. 2015; Sulzman et al. 1982).

While Fig. 2a shows entrainment ranges and phases for zeitgeber signals with a square-wave-like waveform as used in the laboratory, entrainment to light–dark cycles similar to those observed under natural conditions have been studied in (Schmal et al. 2020).

##### Intrinsic oscillator properties affect seasonal entrainment

Entrainment characteristics of the circadian system do not rely only on properties of the zeitgeber signal and the internal period $\tau$ as discussed in the previous paragraphs but also on other intrinsic properties of the circadian clock such as the amplitude, radial relaxation rate, waveform, or twist (i.e., the dependence of the internal period on amplitude). Modeling approaches have been used to show that increasing amplitudes and radial relaxation rates make an oscillator more resistant toward entrainment in comparison to clocks with relatively small amplitudes and relaxation rates (Lakin-Thomas et al. 1991; Abraham et al. 2010). The finding that collective amplitudes and relaxation rates increase due to resonance effects in ensembles of interacting clocks (Abraham et al. 2010; Bordyugov et al. 2011; Schmal et al. 2018) has been used to explain why strongly coupled systems, such as the mammalian core pacemaker, the suprachiasmatic nucleus (SCN), have a narrow entrainment range, while putatively weakly coupled systems like lung or heart tissue rather behave like a weak clock and entrain to more extreme zeitgeber periods (Abraham et al. 2010). This interpretation is further strengthened by the fact that pharmacological decoupling of SCN neurons by MDL or TTX leads to a better entrainability of cultured SCN slices subject to temperature cycles (Abraham et al. 2010) as well as the observation that a faster recovery from jet-lag is observed for mice lacking receptors for the coupling agent arginine vasopressin (AVP) (Yamaguchi et al. 2013). Along these lines, it has been proposed that genetic redundancy within the molecular regulatory network underlying the mammalian circadian rhythm generation strengthens the clock and, thus, leads to narrow entrainment ranges (Erzberger et al. 2013).

##### Bifurcations affect seasonal entrainment

Bifurcations are defined by qualitative changes of systems dynamics due to variations of an internal or external parameters. Many of such qualitative changes in the systems dynamics upon parameter variations have been described for circadian clocks of different organisms. For example in mammals, the dissociation of a single activity band into two bands, termed splitting or frequency doubling, has been observed as a response to changes in zeitgeber properties such as an increasing light intensity under constant conditions (Pittendrigh and Daan 1976b). A transition from self-sustained to damped oscillations has been reported for circadian KaiC rhythms in cyanobacteria after reducing the ambient temperature below 18.6 °C (Murayama et al. 2017).

**Fig. 3:**
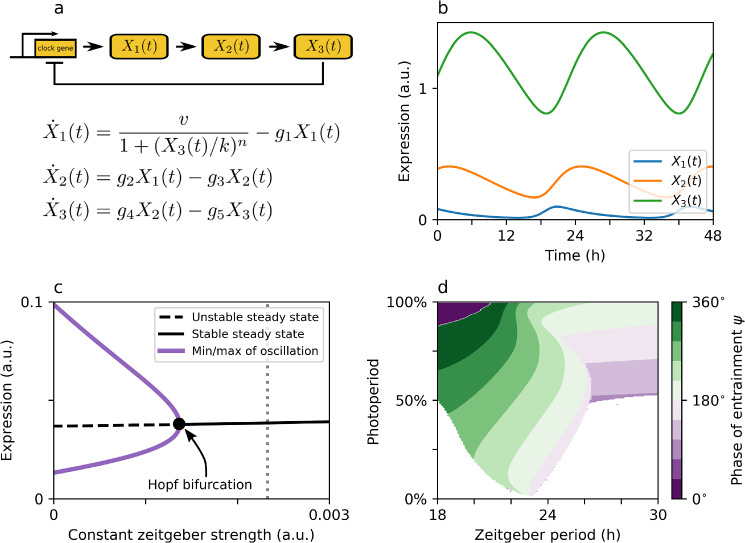
Intrinsic oscillator properties govern seasonal entrainment characteristics.** a** The Goodwin oscillator is considered a blueprint for models of molecular negative feedback loops. We assume that the (square-wave) zeitgeber signal affects the negative feedback loop by an additive term to the $X_1$-variable.** b** Example oscillations under free-running conditions for a parameter set that leads to self-sustained oscillations. Same parameters as those underlying Fig. 8 of (Ananthasubramaniam et al. 2020) have been used.** c** For an increasing constant zeitgeber strength (e.g., constant light of increasing intensity), the system eventually changes its qualitative behavior through a Hopf bifurcation and looses its ability to self-sustain the oscillations.** d** For large zeitgeber intensities, the bifurcation shown in panel (**c**) translates into a broad entrainment range under long photoperiods similar to the behavior of damped oscillators. Panels** c**,** d** are adapted from Fig. S5a and Fig. 8a of (Ananthasubramaniam et al. 2020), respectively (under CC BY 4.0 license)

Such changes in qualitative behavior of circadian oscillator properties will have an impact on the entrainment characteristics as recently reported in a mathematical study using the Goodwin model (Ananthasubramaniam et al. 2020). The Goodwin oscillator is a generic model of a delayed negative feedback loop where the final product $X_3$ of a three-component activatory chain inhibits the production of the first component $X_1$ (Goodwin 1965); see Fig. 3a, b. It fulfills all necessary requirements to produce self-sustained oscillations, such as a negative feedback, non-linearity, as well as delay, and has a long tradition of being applied in modeling circadian clocks (Ruoff et al. 2001; Gonze and Ruoff 2021). Assuming that light enters the model as an additive term to the $X_1$ variable, one observes that increasing constant light finally drives the system to a dampened regime through a Hopf bifurcation; see Fig. 3c. Damped oscillators can be entrained much more easily to rhythmic zeitgeber signals in comparison to a strong self-sustained clock (Bain et al. 2004; Gonze et al. 2005). Thus, forcing the Goodwin oscillator with a zeitgeber intensity that corresponds to a value that would drive the system to a damped regime under constant conditions (e.g., gray dotted line in Fig. 3c) leads to broadening of the entrainment region under long photoperiods (Fig. 3d). A similar behavior that relies on the bifurcation structure of the underlying molecular feedback loop has been observed in *Neurospora crassa* subject to temperature entrainment, where increasingly damped oscillations for decreasing temperatures (Liu et al. 1997b) translate into an experimentally observed broader entrainment range under short thermoperiods (Burt et al. 2021).

#### Contextual models

While conceptual models as described so far focus on generic properties of the oscillatory circadian system, contextual models try to understand properties and design principles of the circadian clock with respect to organism, tissue, or cell-type-specific details. In a simplified schematic view of the circadian clock, known as Eskinogram, the clock system can be divided into an input pathway that integrates external zeitgeber signals, the circadian core pacemaker, and output pathways or subordinate clocks. Contextual models have been proposed for all of these three regulatory layers and have been subsequently used for studying entrainment properties of the circadian clock.

For the human circadian system, *Kronauer, Forger, and Jewett* proposed a model for the biochemical processes that pre-process and convert light information for the circadian core pacemaker and couple this model of the retinal light input pathway to a conceptual van der Pol oscillator model (Kronauer et al. 1999; Forger et al. 1999) that has a long tradition of being used in circadian clock modeling (Wever 1964, 1972; Kronauer et al. 1982). The model accurately predicts experimental light stimulus data (Forger et al. 1999) and has been subsequently used to study general principles underlying circadian (seasonal) entrainment (Creaser et al. 2021), entrainment under field conditions (Stone et al. 2020), interactions between circadian rhythms and homeostatic sleep drive (Phillips et al. 2010), as well as the re-entrainment time (i.e., jet-lag duration) for traveling between regions of different season that occurs for north- or southward directions (Diekman and Bose 2018).

A large variety of detailed contextual molecular models of the intracellular regulatory feedback loops have been proposed for various model organisms including *Neurospora crasssa* (Hong et al. 2008), the small flowering plant *Arabidopsis thaliana* (Locke et al. 2006; Fogelmark and Troein 2014; De Caluwe et al. 2016), the fruit fly *Drosophila melanogaster* (Leloup and Goldbeter 1998), and mammals (Forger and Peskin 2003; Relógio et al. 2011; Korencic et al. 2012). Such contextual models helped to reveal design principles underlying circadian rhythm generation at the intracellular level but have also been used to better understand which model architectures of the clock and its light input pathways allow for a proper entrainment across different seasons (Thommen et al. 2015; De Caluwé et al. 2017). Along these lines, Troein et al. (2009) used an evolutionary optimization approach to find clock models that best adapt to various photoperiods and weather-induced stochasticity.

Finally, detailed molecular contextual models have been proposed to understand design principles underlying the processing of circadian signals as perceived by the output pathways of the circadian clock. This includes driven feedback loops subordinate to the core pacemaker (Schmal et al. 2013) as well as output pathways controlling starch metabolism in plants (Seaton et al. 2014), liver metabolism in mammals (Woller et al. 2016), or those triggering seasonal responses such as flowering time in plants (Salazar et al. 2009) or photoperiodic responses in mammals (Ebenhöh and Hazlerigg 2013).

### Photoperiodic encoding

The circadian system does not only passively react to changes in photoperiods by tuning its entrained amplitude and phase $\psi$ as a response to seasonal variations in zeitgeber signals as discussed in the section *Seasonal entrainment*, it also plastically changes its internal properties as a response to the previously experienced light schedule. In mammals, the suprachiasmatic nucleus (SCN) has been identified as the central circadian pacemaker. Located in the anterior hypothalamus, it consists of approximately $10^4$ bilaterally distributed neurons and is a remarkable example of localized brain functionality. Ablation of the SCN leads to a suspension of circadian behavioral activity and its re-transplantation restores such rhythmicity within approximately one week (Ralph et al. 1990). Based on the expression of neuropeptides, the SCN is often dichotomized into a ventro-lateral (core) and a dorso-medial (shell) part. Experimentally observed re-organizations of spatio-temporal pattern formation between the core and shell part of the SCN in response to changing environmental photoperiods have been supposed as an internal representation of seasons (Coomans et al. 2015). These findings are in favor of the original hypothesis of *Erwin Bünning* from 1936 that the circadian clock, in addition to being a daily clock also serves as a seasonal timing device by measuring the day-length internally (Bünning 1936). Evidence from knockout mice lacking the neurotransmitter VIP that loose their ability to encode seasonal information suggests that inter-cellular coupling between SCN neurons is essential for proper photoperiodic encoding in mammals (Lucassen et al. 2012).

Prior to discussing principles underlying seasonal encoding, we discuss mathematical approaches that help to understand how coupling leads to the experimentally observed spontaneous synchronization or self-entrainment in networks of interacting clocks.

#### Precision through coupling

It has been shown that inter-cellular communication between SCN neurons, relying on neuropeptidergical, synaptic, and gap-junctional couplings, is quintessential for generating the remarkable precision observed at the tissue and behavioral level (Yamaguchi et al. 2003; Herzog et al. 2004). Single cellular oscillations of widely dispersed SCN neurons have been reported to show a relatively large standard deviation of $\sigma = 1.28\hbox {h}$ in comparison to $\sigma =0.32\hbox {h}$ and $\sigma =0.13\hbox {h}$ at the tissue explant and organism level, respectively (Herzog et al. 2004). Analogous to the physical separation of SCN neurons, pharmacological suspension of synaptic couplings by tetrodotoxin (TTX) reversibly leads to larger phase distributions of clock gene oscillations in organotypic SCN slices, i.e., a less precise clock (Yamaguchi et al. 2003; Abel et al. 2016; Schmal et al. 2018). Besides coupled SCN neurons in the mammalian core pacemaker, communicating circadian clocks have been described in and between peripheral tissues as well. Density-dependent rhythmicity in cultured fibroblasts (Noguchi et al. 2013) and hepatocytes (Guenthner et al. 2014) is indicative of coupling (Micklem and Locke 2021). In addition, mathematical modeling suggests that the choroid plexus, a non-neuronal brain tissue that harbors a robust clock more precise than the SCN gains its high precision through nearest-neighbor gap junctional coupling (Myung et al. 2018).

##### A conceptual phase oscillator approach

**Fig. 4:**
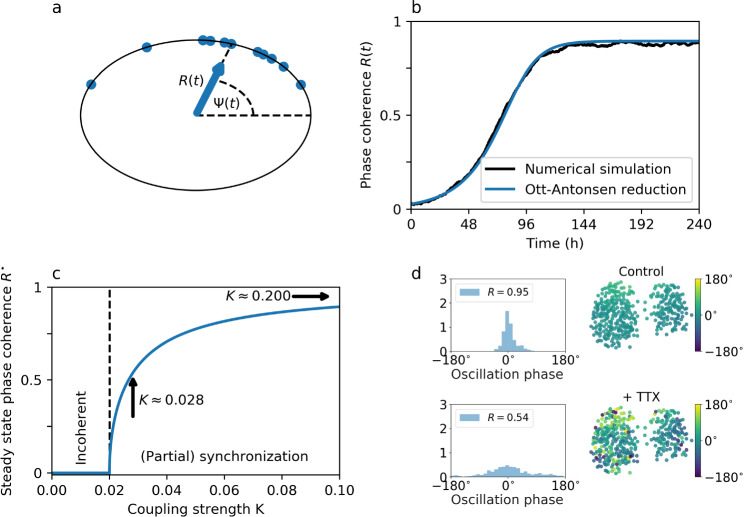
Precision through coupling. a) Phase distributions in large ensembles of clocks can be conveniently visualized on the unit circle of radius 1. The global order parameter $R e^{i \, \Psi }$, depicted as a blue arrow, conveniently summarizes macroscopic properties of the ensemble. While the phase coherence *R* is given by the length of the arrow, the average phase $\Psi$ defines the position of the arrow head similar to the clock hands of a classical mechanical clock. b) The Ott–Antonsen reduction method faithfully reproduces numerical results in an ensemble of uniformly coupled Kuramoto oscillators with unimodally (Cauchy–Lorentz) distributed intrinsic frequencies $\omega _i=\frac{2\pi }{\tau _i}$. c) For large enough coupling strength, i.e., $K>2\gamma$, oscillators show spontaneous synchronization. Subsequently, the phase coherence increases, i.e., the phase-spread decreases, for increasing coupling strength. Here, a value of $\gamma =0.01$ has been used which approximately corresponds to the experimentally observed standard deviation of $\sigma = 1.28 h$ for widely dispersed SCN neurons, estimated in the period domain (Herzog et al. 2004). The dashed black line denotes the critical coupling strength $K_c=2\gamma$. d) Representative oscillation phase distribution of PER2::LUC reporter gene expression for individually tracked SCN neurons of cultured SCN slices under control conditions (top) and during application of tetrodotoxin (bottom). Phases are shown in a histogram (left) and at their original positions within the SCN slice (right). Original data have been obtained from (Abel et al. 2016) and oscillation phases have been determined as previously described (Schmal et al. 2018). Arrows in panel (c) point to coupling strength $K\approx 0.028$ and $K\approx 0.200$ that lead to a phase coherence of $R=0.54$ and $R=0.95$ as observed for the exemplary experimental data under control and TTX conditions as shown in panel (d), respectively

A natural description of oscillator phase dynamics in a system of coupled clocks like the SCN without explicit consideration of the intricate molecular details of intracellular rhythm generation is given by Kuramoto models (Kuramoto 1975; Strogatz 2000; Kuramoto 2003)

14 $$\begin{aligned} \frac{d\theta _i}{dt} = \omega _i + \sum _{j=1}^N K_{i,j} \sin (\theta _j-\theta _i), \end{aligned}$$

which are a generalization of the coupled phase oscillator model (8), described in the section “Phase oscillator models”. Here, $\theta _i$ is the phase of oscillator *i*, *N* the number of total oscillators in the network, $\omega _i=\frac{2\pi }{\tau _i}$ the angular velocity of oscillator *i*, $K_{i,j}$ the coupling strength of the interaction from oscillator *j* onto oscillator *i*, and $\sin (\theta _j-\theta _i)$ the corresponding interaction or coupling function. A convenient way to study the collective dynamics in such an ensemble of coupled oscillators is to introduce the global order parameter $R e^{i \, \Psi }=\frac{1}{N}\sum _{j=1}^{N} e^{i\,\theta _j}$ with *i* being the complex element. Here, *R* and $\Psi$ are the phase coherence and mean phase in the ensemble of clocks, respectively, and thus describe the macroscopic state of the network dynamics, see Fig. 4a.

In the context of circadian clocks, this conceptual phase oscillator model as given by Eq. (14) has been used to analyze how the circadian free-running period observed at the behavioral level is determined by the ensemble average of the individual clock cell periods, measured for widely dispersed SCN neurons in wild type as well as heterozygous and homozygous tau-mutant Syrian hamsters (Liu et al. 1997a).

##### Low-dimensional representation of phase oscillator network dynamics

Recent mathematical advances tremendously facilitate the analysis of the complex network dynamics given by Eq. (14) and thus to infer properties of single cellular parameters and coupling topologies of networked circadian clocks. Similar to the theory of statistical mechanics in physics where macroscopic variables, such as temperature, entropy, or pressure of a given system are derived from the microscopic dynamics in a large ensemble of particles, *Edward Ott* and *Thomas M. Antonsen* proposed a method that allows to describe the complex dynamics in a large ensemble of *N* coupled oscillators by low- dimensional representations using macroscopic variables such as the phase coherence *R* or mean phase $\Psi$ (Ott and Antonsen 2008, 2009).

Applying the *Ott-Antonsen* reduction method to our prototypical example of mean-field coupled phase oscillators as given by Eq. (14) under the additional assumption that the internal frequencies $\omega _i$ follow a unimodal (Cauchy–Lorentz) distribution of periods, allows to essentially reduce the dynamics of the *N*-dimensional set of equations (14) to a simplified one-dimensional description of the temporal evolution of the ensemble’s phase coherence:

15 $$\begin{aligned} \frac{dR}{dt} = \frac{K}{2} R (1-R^2)-\gamma R, \end{aligned}$$

where $\gamma$ relates to the spread of the distribution of cell autonomous periods $\tau _i$, see also (Ott and Antonsen 2008). Figure 4b illustrates the good accordance between the numerical simulations of full model (14) and the reduced dynamics given by (15). From this low-dimensional representation, steady-state dynamics (fixed-points) after the decay of transients ($t \rightarrow \infty$) can be readily inferred by searching for the values of the global phase coherence $R(t)=R^{\star }$, such that the right-hand side of Eq. (15) equals zero, i.e., no further changes in the dynamics occur. It straightforwardly follows that no synchronization between the oscillators occurs (i.e., the incoherent state $R^{\star }=0$ is stable) in cases where the coupling strength *K* does not exceed two-times the frequency spread $\gamma$ (i.e., $K<2\gamma$). Synchronized ensemble dynamics emerge for large enough coupling strength $K>2\gamma$, i.e., the so-called coherent network state $R^{\star }=\sqrt{1-\frac{2\gamma }{K}}$ becomes stable, with an increasing phase coherence *R* for increasing coupling strength *K* or decreasing spread of internal period distribution $\gamma$; see Fig. 4c.

Such predictable changes in the distributions of individual oscillator phases and periods but also amplitudes due to resonance effects have been used to quantify the relative strength of inter-cellular coupling in the mammalian circadian core pacemaker (Schmal et al. 2018) as well as peripheral clocks such as cultured U-2 OS cells (Finger et al. 2021). To give an example, we re-analyze the distributions of clock gene expression phases, measured by a PER2::LUC reporter for cultured SCN slices under control conditions and during the suspension of synaptic coupling after TTX application as previously described; see Fig. 4d for representative snapshots. By comparing the experimentally obtained phase coherences with findings from our modeling approach (see *arrows* in Fig. 4c), one can show that the phase distribution under TTX treatment corresponds to a relatively weakly coupled network state, similar to findings in Schmal et al. (2017, 2018).

Extensions of this model such as an (Ott–Antonsen reduced) uniformly coupled network of circadian clocks that is, in addition, driven by a 24 h sinusoidal light–dark cycle, have been used to study cross-time-zone traveling and to reveal principles underlying the observed difference in re-synchronization duration between eastward and westward travel (Lu et al. 2016).

Please note that a technical limitation of the *Ott–Antonsen* approach is that in order to be able to derive a reduced macroscopic model such as (15) from the *N*-dimensional full set of microscopic equations, one usually requires the assumption that the intrinsic frequencies $\omega _i$ follow a Cauchy–Lorentz distribution or the superposition of multiple Cauchy–Lorentz distributions in case of oscillator communities with different (mean) periods in each community (Martens et al. 2009; Skardal 2019). It has been found that a related method, the $m^2$ ansatz, yields quantitatively better results for populations of coupled clocks having frequency distributions with exponential tails such as Gaussians, even though the qualitative dynamics obtained from the $m^2$ or *Ott–Antonsen* approach might be comparable (Hannay et al. 2018).

##### Models of coupled circadian clocks reveal principles underlying efficient synchronization

During the last decades, mathematical models of networked circadian clocks have been proposed to study the effect of network topologies and intrinsic oscillator properties on synchronization properties in ensembles of coupled clocks. It has been shown that increasing the number of locally coupled van der Pol oscillators as a model for the SCN leads to the emergence of a stable overall rhythm and a higher resistance against noise (Achermann and Kunz 1999; Kunz and Achermann 2003). Networks of coupled conceptual Poincaré (see Eqs. (12), (13)) or Goodwin (see Fig. 3a) oscillators have been used to study the effect of network topology (Gu and Yang 2016), intrinsic oscillator properties (Gu et al. 2018, 2019), the fraction of light-perceiving neurons (Gu et al. 2014), and zeitgeber waveforms (Zheng et al. 2022) on the overall entrainment capability of the SCN to external zeitgeber cues.

Interestingly, studies using mean-field coupled Goodwin oscillators suggest that bifurcations, i.e., qualitative changes in the dynamics of individual SCN neurons, similar to those described in the paragraph *Bifurcations affect seasonal entrainment* can achieve an efficient synchronization between SCN neurons. For certain mean-field coupling strengths the average neurotransmitter concentration can drive the individual SCN neurons to a dynamical regime with damped oscillations which, in turn, eventually allows them to entrain easier to the mean-field rhythm of the coupling agents (Gonze et al. 2005; Locke et al. 2008).

While all models described in this section mainly focused on principles that determine the synchronizabilty in ensembles of interacting oscillators, we focus in the next section on how plastic network re-organizations allow to explain the experimentally observed photoperiodic encoding in the mammalian core pacemaker.

#### Photoperiodic encoding through network re-organizations

**Fig. 5:**
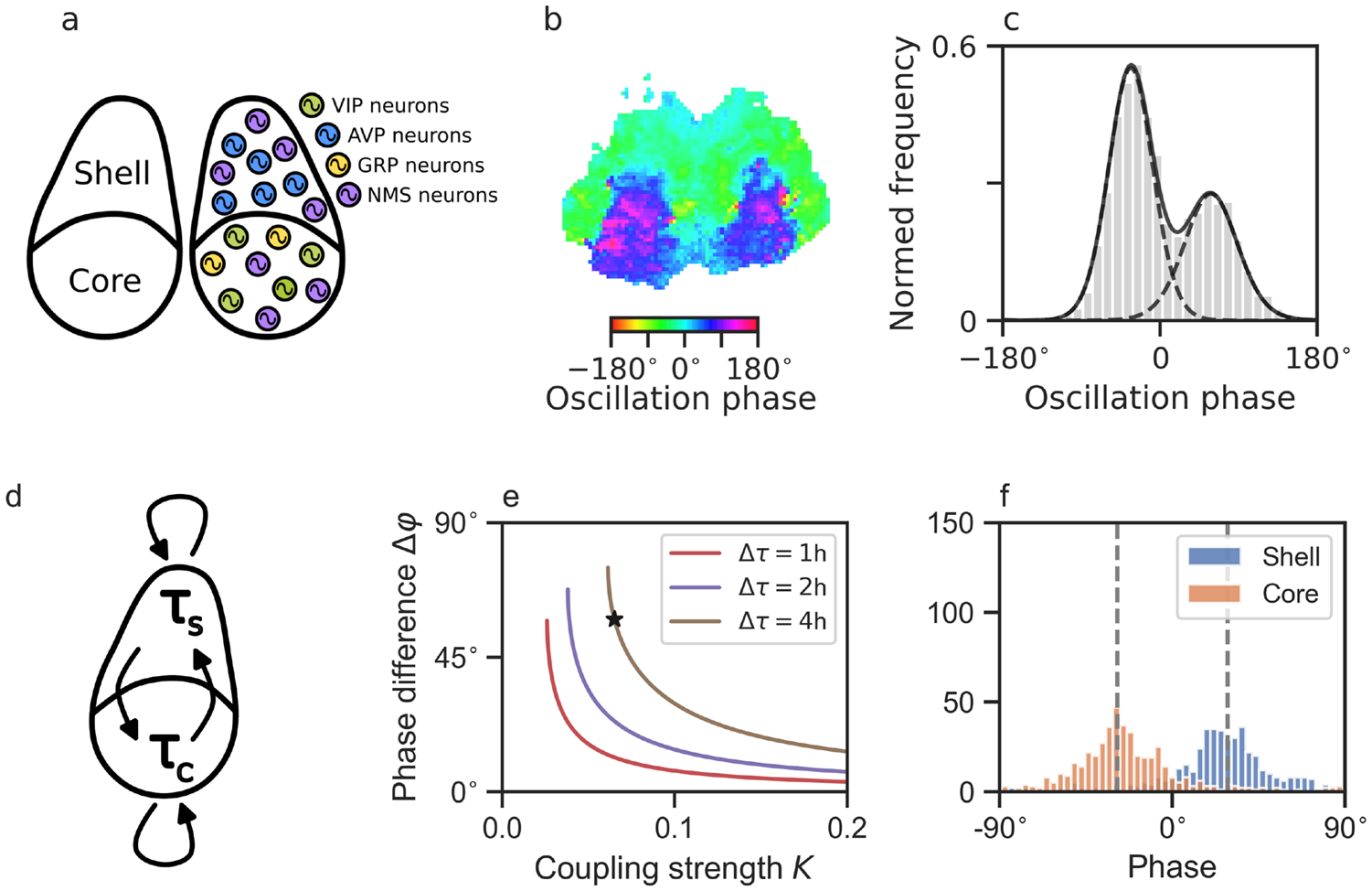
Photoperiodic encoding. **a** Schematic drawing of region-specific neuropeptide expression within the SCN. **b** Emergence of spatial phase clustering in cultured SCN slices from mice entrained to extremely long photoperiods of LD20:4. **c** Histogram of phase values depicted in panel (**b**) shows a bimodal distribution. Bold and dashed black lines denote a bimodal composite van Mises distribution and the underlying unimodal distributions, respectively, fitted to the histogram data (gray bars). Fitting reveals that the mean phases in the core and shell are separated by $90^{\circ {}}$ after entrainment to light–dark cycles of extremely long photoperiods (LD20:4). Data have been obtained from Evans et al. (2013) and analyzed as previously reported (Schmal et al. 2017). **d** Schematic drawing of an SCN model, constituted of two groups of interacting oscillators, i.e., core and shell neurons, under the assumption that intracellular oscillators of core and shell neurons follow intrinsic frequency distributions of different mean; see “Materials and Methods” for further model details. **e** Increasing period differences $\Delta \tau = \tau _C - \tau _S$ as well as decreasing coupling strength *K* between the core (ventral) and shell (dorsal) neurons can lead to an increasing gap between the oscillation phases of core and shell neurons. Color-coded bifurcation diagrams of the Ott–Antonsen reduced system given by Eqs. (6), (7) of the section “Materials and Methods” have been obtained by XPP-Auto. **f** Ott–Antonsen reduced dynamics faithfully reproduce the behavior of numerical simulations of the full set of equations, compare *dashed lines* representing the steady-state phases in the core and shell neurons in the low-dimensional Ott–Antonsen reduced representation with the corresponding numerically obtained phase distributions depicted by bar plots. Simulations shown in (**f**) correspond to the parameter values depicted by the *star* in (**e**), i.e., a coupling strength of $K=0.065$, a period difference of $\Delta \tau = 4h$ corresponding to $\tau _C = 26\hbox {h}$ and $\tau _S = 22\hbox {h}$ as well as a frequency spread of $\gamma = 0.01$ as used in Fig. 4b, c

The SCN is an anatomically heterogeneous tissue. While the shell or dorso-medial part of the SCN expresses mainly arginin vasopressin (AVP), the core or ventro-lateral part expresses vasoactive intestinal peptide (VIP), and gastrin releasing peptide (GRP); see Fig. 5a for a schematic drawing. The neurotransmitter $\gamma$-aminobutyric acid (GABA) has been shown to be expressed in both subregions and can act as a de-synchronizing or synchronizing agent, depending upon the system state of the SCN (Evans et al. 2013; Freeman Jr. et al. 2013; Myung et al. 2015). Depending upon developmental stages and environmental conditions, the SCN can show complex spatio-temporal patterns such as phase waves or phase-clustering (Quintero et al. 2003; Evans et al. 2011; Fukuda et al. 2011; Myung et al. 2012). These phase organizations rely on the previously applied light-schedule and photoperiod and it is thus believed that the network-level organization constitutes an internal representation of seasons. While small regional phase differences of PER2::LUC reporter construct rhythms are observed in cultured SCN slices after equinoctial 24 h light–dark schedules (LD12:12), long-day entrainment with 20 h of light and 4 h of darkness (LD20:4) leads to phase clusters and region-specific phase differences of up to 12 h (Evans et al. 2011, 2013); see Fig. 5b, c. Likewise, at the electrophysiological level, in vivo neuronal activity profiles in freely-moving mice are compressed under short-day in comparison to long-day entrainment (VanderLeest et al. 2007).

What are the design principles underlying photoperiodic encoding? A macroscopic state such as the occurrence of phase waves or phase clusters in systems of networked oscillators such as the SCN is generally determined by both intrinsic oscillator properties as well as the coupling topology of the network. Indeed, using a conceptual *Kuramoto* model approach, one can show that the occurrence of phase clusters could (i) either rely on a globally uniformly coupled population of clocks where two disjoint communities of SCN neurons such as those in dorsal and ventral region have distinct intrinsic free-running periods (Martens et al. 2009; Zhang et al. 2020) being consistent with the finding that periods in the core and shell differ (Noguchi et al. 2004; Myung et al. 2012) or (ii) rely on the re-arrangements of the coupling strength between those regions (Hong and Strogatz 2011; Sonnenschein et al. 2015; Skardal 2019). To give an example, we study a system of coupled Kuramoto oscillators as given by Eq. (14) that is functionally separated into two groups, related to the core (ventral) and shell (dorsal) neurons of the SCN, under the assumption that intrinsic frequencies of core and shell neurons follow distributions with different mean values; see Fig. 5d and section “Materials and Methods” for further model details. For the sake of simplicity, we assume that the fraction of core and shell neurons is equal, that the spread of frequencies (or periods) is identical in the core and shell part of the SCN and that the inter- and intra-group coupling is of identical strength *K*. Using these assumptions, one can observe that, indeed, an increasing period difference between the core and shell neurons as well as a decreasing coupling strength *K* leads to an increasing gap between the oscillation phases of the core and shell neurons (Fig. 5e, f). More complicated models, including asymmetries such as different fractions of core and shell neurons, a varying spread of frequency distributions in core and shell neurons, or an asymmetric strength of inter- and intra-group coupling, may give rise to symmetry breaking, more complex dynamics and opens the possibility to further tune the phase separation between the core and shell part of the SCN. Along these lines, a modeling study suggests that a relatively stronger coupling from the core (ventral) to the shell (dorsal) part of the SCN in comparison to the opposite coupling direction is consistent with the experimentally observed re-synchronization dynamics after a reversible pharmacological decoupling of cell-to-cell communication (Taylor et al. 2017).

Extensions of above-described *Kuramoto* models have been used to study the SCN under different entrainment conditions. The SCN receives light information through the retinohypothalamic tract via melanopsin-containing intrinsically photosensitive retinal ganglion cells (ipRGCs) (Hattar et al. 2002). Classical rod and cone photoreceptors contribute under certain conditions (Walmsley et al. 2015). While previous works suggested that light-responsive neurons are mainly found in the ventral part of the SCN (Meijer and Schwartz 2003), recent studies revealed that ipRGCs innervate VIP, GRP, and AVP neurons in the ventral and dorsal parts of the SCN with the vast majority of innervations found in the ventral region (Fernandez et al. 2016). A two-community model with the ventral (core) part of the SCN being entrained by light signals in combination with an Ott–Antonsen reduction has been used to study how the functional separation into core and shell affects the tissues upper and lower limits of entrainment and allows to explain the experimentally observed dissociation phenomena (Goltsev et al. 2022), i.e., the emergence of a second rhythm in SCN activity and behavior with a different period in comparison to the entrainment cue. Hannay et al. (2020) revealed that the photoperiod-induced adjustment of coupling strength between the ventral and dorsal part of the SCN under the assumption that only the ventral part receives light information yields a mutually consistent unifying explanation of three light-mediated circadian effects, namely, (i) the increasing phase gap under long photoperiods, (ii) the experimentally observed photoperiod-depending aftereffects (Pittendrigh and Daan 1976a), as well as (iii) the apparently counter-intuitive observation that the amplitude of phase response curves with respect to light perturbations decreases for mammals entrained to long photoperiods (vanderLeest et al. 2009; Ramkisoensing et al. 2014). Similar findings have been obtained by a purely numerical study in (Gu et al. 2016).

The functional separation of the SCN into two regionally disjoint sub-groups, i.e., a core and a shell part, gave rise to modeling approaches using the assumption that the two communities essentially act like two separated oscillators mutually coupling to each other (Myung and Pauls 2018). This approach is similar to the original proposition of *Pittendrigh*, *Daan* and *Berde*, assuming that (at least) two autonomously oscillating coupled oscillators, termed morning (M) and evening (E) oscillators, underlie the rhythm generation in the mammalian pacemaker which helped to explain the experimentally observed splitting of behavioral rhythms into two components (Pittendrigh and Daan 1976b; Daan and Berde 1978; Helfrich-Förster 2009). Along these lines conceptual network models where functionally separated groups within a larger network of coupled oscillators are associated with mutually coupled *single* oscillators have been used to study several phenomena in chronobiology including photoperiodic encoding (Myung and Pauls 2018) and aftereffects (Azzi et al. 2017) in the SCN, exposure to skeleton photoperiods (Flôres and Oda 2020), splitting phenomena (Indic et al. 2008; Oda and Friesen 2002), the experimentally observed transient dynamical dissociation between different clock genes of the intracellular transcriptional-translational feedback loops after external perturbations (Schmal et al. 2019), as well as the synchronization of circadian rhythms of different brain areas such as the area postrema, the nucleus of the solitary tract, and the ependymal cells surrounding the fourth ventricle (Ahern et al. 2023).

## Concluding remarks

In addition to experimental advances, mathematical modeling contributed to understand principles of circadian entrainment, intracellular rhythm generation through transcription–translation feedback loops (TTFL) and non-TTFL mechanisms, as well as the synchronization of interacting circadian entities. Theoretical approaches, mathematical modeling, and numerical simulations can help to understand complex dynamics and counter-intuitive results that may otherwisely be hard to grasp.

In this review, we explored various mathematical approaches to understand seasonal entrainment and photoperiodic encoding ranging from individual clocks entrained by an external zeitgeber to complex networks of coupled oscillators. Recent theoretical advances such as the *Ott-Antonsen* or $m^2$ approach that facilitate the analysis of network dynamics are introduced and discussed in the context of circadian systems. In the physics literature, these approaches have been used to study numerous realizations of coupled oscillator networks with different oscillator properties and network topologies, yielding a large potential to utilize these theoretical advances in future chronobiological studies.

Throughout the review, we generally focus on two extreme cases of networked oscillatory systems. While the entrainment studies discussed in the section “Seasonal entrainment” study dynamical properties of a single oscillator driven by an external zeitgeber signal, synchronization between SCN neurons underlying photoperiodic encoding as discussed in the section “Photoperiodic encoding” typically consists of thousands of coupled clocks, allowing for a proper analysis using averaging methods such as the Ott–Antonsen approach. In contrast, dynamics of mesoscopic systems such as the circadian clock in flies are much harder to study and further theoretical advances are needed.
